# DNA methylation dynamics during early plant life

**DOI:** 10.1186/s13059-017-1313-0

**Published:** 2017-09-25

**Authors:** Daniel Bouyer, Amira Kramdi, Mohamed Kassam, Maren Heese, Arp Schnittger, François Roudier, Vincent Colot

**Affiliations:** 1 Institut de Biologie Moléculaire des Plantes du CNRS - UPR2357, Université de Strasbourg, Strasbourg, France; 2 Institut de Biologie de l’Ecole Normale Supérieure, CNRS UMR 8197–INSERM U 1024, F-75230 Paris, France; 3 Present address: Nestlé Institute of Health Sciences, Functional Genomics, Lausanne, Switzerland; 4 Department of Developmental Biology, University of Hamburg, Biozentrum Klein Flottbek, Hamburg, Germany; 5 Present address: Laboratoire Reproduction et Développement des Plantes, Univ Lyon, ENS de Lyon, UCB Lyon 1, CNRS, INRA, F-69342 Lyon, France

**Keywords:** DNA methylation, RdDM, Transposable elements, Embryogenesis, Embryo-seedling transition

## Abstract

**Background:**

Cytosine methylation is crucial for gene regulation and silencing of transposable elements in mammals and plants. While this epigenetic mark is extensively reprogrammed in the germline and early embryos of mammals, the extent to which DNA methylation is reset between generations in plants remains largely unknown.

**Results:**

Using *Arabidopsis* as a model, we uncovered distinct DNA methylation dynamics over transposable element sequences during the early stages of plant development. Specifically, transposable elements and their relics show invariably high methylation at CG sites but increasing methylation at CHG and CHH sites. This non-CG methylation culminates in mature embryos, where it reaches saturation for a large fraction of methylated CHH sites, compared to the typical 10–20% methylation level observed in seedlings or adult plants. Moreover, the increase in CHH methylation during embryogenesis matches the hypomethylated state in the early endosperm. Finally, we show that interfering with the embryo-to-seedling transition results in the persistence of high CHH methylation levels after germination, specifically over sequences that are targeted by the RNA-directed DNA methylation (RdDM) machinery.

**Conclusion:**

Our findings indicate the absence of extensive resetting of DNA methylation patterns during early plant life and point instead to an important role of RdDM in reinforcing DNA methylation of transposable element sequences in every cell of the mature embryo. Furthermore, we provide evidence that this elevated RdDM activity is a specific property of embryogenesis.

**Electronic supplementary material:**

The online version of this article (doi:10.1186/s13059-017-1313-0) contains supplementary material, which is available to authorized users.

## Background

DNA methylation is an epigenetic modification with key roles in the control of genome activity in mammals and plants. It is involved in the transcriptional silencing of transposable elements (TEs), thus contributing to the preservation of genome integrity, as well as in the regulation of specific genes, such as those subjected to genomic imprinting [1, 2]. Despite these general similarities, there are many differences in function and mechanism of DNA methylation between mammals and plants. Whereas mammals mainly methylate cytosines at symmetrical CG sites, plants also methylate cytosines at CHG (H = A, T, or C) and CHH sites, although to a lesser extent than at CG sites [1]. Also, while mammals methylate genes and TE sequences equally [1], plants methylate only some genes and this methylation is usually restricted to CGs located within the gene body while TE sequences tend to be methylated at most, if not all, of their CG, CHG, and CHH sites [3, 4].

Plants and mammals also differ in the extent to which DNA methylation is reprogrammed at each generation. In mammals, the early embryo and the male as well as the female primordial germlines undergo extensive loss of DNA methylation, which together with other epigenome resetting events ensures that totipotency is re-established at each generation [5]. In contrast, flowering plants, which undergo double fertilization of the egg cell (EC) and the central cell (CC) to produce the embryo and the endosperm within seeds, do not appear to show such extensive DNA methylation reprogramming, except in the endosperm. Specifically, work in Arabidopsis, maize, and rice has shown that the maternal genome is globally hypomethylated in the endosperm and it is thought that this hypomethylation originates from active DNA demethylation in the CC as well as from reduced expression of the main DNA methyltransferases (MTases) [6–10]. In contrast, there is no detectable demethylation activity in the EC although cytological studies revealed a weakening in the DNA methylation signal [8, 11, 12].

On the paternal side, there is active DNA demethylation in the vegetative nucleus (VN) of pollen that is, however, limited to only a few TE sequences [6, 13]. Lastly, genome-wide studies revealed similar DNA methylation patterns in the early embryo and adult aerial tissues [11]. Nonetheless, loss of DNA methylation over TE sequences in the endosperm and the VN has been proposed to serve as a source of small interfering RNAs (siRNAs) that would trigger RNA-directed DNA methylation (RdDM) in the embryo [14, 15], thus ultimately contributing to the reinforcement of DNA methylation across generations [16, 17].

In the present work, we show that unlike CG methylation, CHG and CHH methylation is dynamic during embryogenesis and early vegetative development. Most notably, mature embryos show 100% CHH methylation at many sites, which are not observed in seedlings or adult plants. Furthermore, these highly methylated TE sequences are hypomethylated in the early endosperm. Our findings reveal an important role for RdDM during embryogenesis, which reaches its maximum in the mature embryo and may serve to protect embryonic cells from the deleterious consequences of TE activity.

## Results

### Mature embryos show CHH hypermethylation

**Fig. 1:**
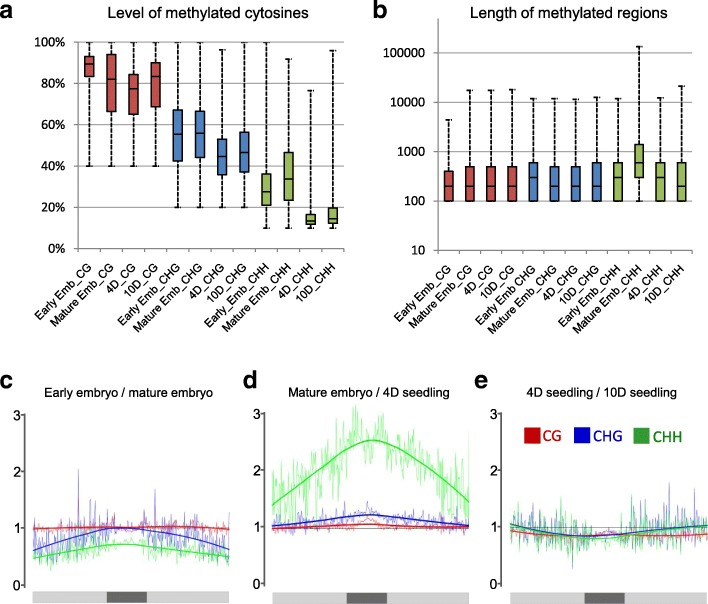
Global methylome dynamics between different developmental stages. **a**, **b** *Box plot* distribution of DNA methylation levels (**a**) and length of MRs (**b**) at different developmental stages (early embryo [Early_Emb], mature embryo [Mature_Emb], four-day-old seedling [4D], ten-day-old seedling [10D]) in the WT for each context. **c**–**e** Methylation ratios of 100 kb windows for CG (*red*), CHG (*blue*), and CHH (*green*), represented as a linear regression curve over chromosome 1 (*gray bar*, pericentromeric region in *dark gray*). Comparison of early embryos (7–9 days after fertilization [11]) vs. mature embryos (**c**), mature embryos vs. four-day-old seedlings (**d**), and four-day-old vs. ten-day-old seedlings (**e**)

To analyze the dynamics of DNA methylation during the early stages of the Arabidopsis life cycle, whole-genome sequencing (WGS) was performed on bisulfite-treated DNA (WGBS) extracted from mature wild-type (WT) embryos as well as from four-day-old and ten-day-old seedlings (Additional file 1: Table S1). Data were then compared with publicly available WGBS data obtained for early embryos (7–9 days post fertilization [11]). At a global level, DNA methylation is highest in mature embryos (7.65% 5mC, vs. 6.8%, 4.48%, and 5.6% 5mC for early embryos, four-day-old, and ten-day-old seedlings, respectively; Additional file 2: Figure S1). These variations in global DNA methylation are mainly due to CHH sites (Fig. 1a, b), which make up more than 44% of all 5mC in mature embryos compared to less than 27% in seedlings (Additional file 2: Figure S1). The global distribution of DNA methylation along chromosomes using 100-kb windows confirms these observations (Fig. 1c–e). It shows, in addition, that the lower CHH methylation in early compared to mature embryos is most pronounced away from the TE-dense, gene-poor, pericentromeric regions, whereas elevated CHH methylation in mature embryos compared to four-day-old seedlings is highest over the pericentromeric regions (Fig. 1c–e; Additional file 2: Figure S2). Consistent with immunocytochemical studies [18, 19], we found lower overall levels of DNA methylation in four-day-old compared to ten-day-old seedlings (Fig. 1a), that is most pronounced in pericentromeric regions and affects all three sequence contexts (Fig. 1e; Additional file 2: Figure S2). These results suggest distinct dynamics of CHH methylation along chromosomes with initiation of elevated levels at the pericentromeric region in early embryos and increasing CHH methylation levels also affecting chromosome arms in mature embryos.

We next investigated methylated regions (MRs), which were defined by considering non-overlapping 100-nt windows. Windows were scored as methylated at CG, CHG, or CHH sites when methylation levels reached at least 40%, 20%, or 10% for the respective window (see “Methods” for further details). This analysis revealed that the average size and level of methylation of CG- and CHG-MRs do not change appreciably from early embryos to ten-day-old seedlings. In contrast, CHH-MRs vary considerably during early plant life, being largest and most methylated in mature embryos (Fig. 1a, b; average size of CHH-MR: > 2 kb vs. < 500 bp).

Differentially methylated regions (DMRs) at the 100-nt window scale were then identified by comparing earlier with later developmental stages and by taking into account an average methylation difference of at least 40% for CG sites and 20% for CHG as well as CHH sites. Windows with three or fewer sites were not considered. Applying this approach, thousands of DMRs are observed for all three contexts and this holds true when comparing any two stages (Additional file 2: Figure S3; Additional file 3: Table S2; Additional file 4: Table S3; Additional file 5: Table S4). Most CG-DMRs correspond to variation in gene-body methylation (Additional file 2: Figure S4) and result from the gain or loss of methylation over one or two CGs only, with no coherent patterns across stages (Additional file 2: Figure S5). These CG-DMRs most likely reflect minor stochastic fluctuations between the different WT lines used to extract DNA [20] and were not analyzed further.

**Fig. 2:**
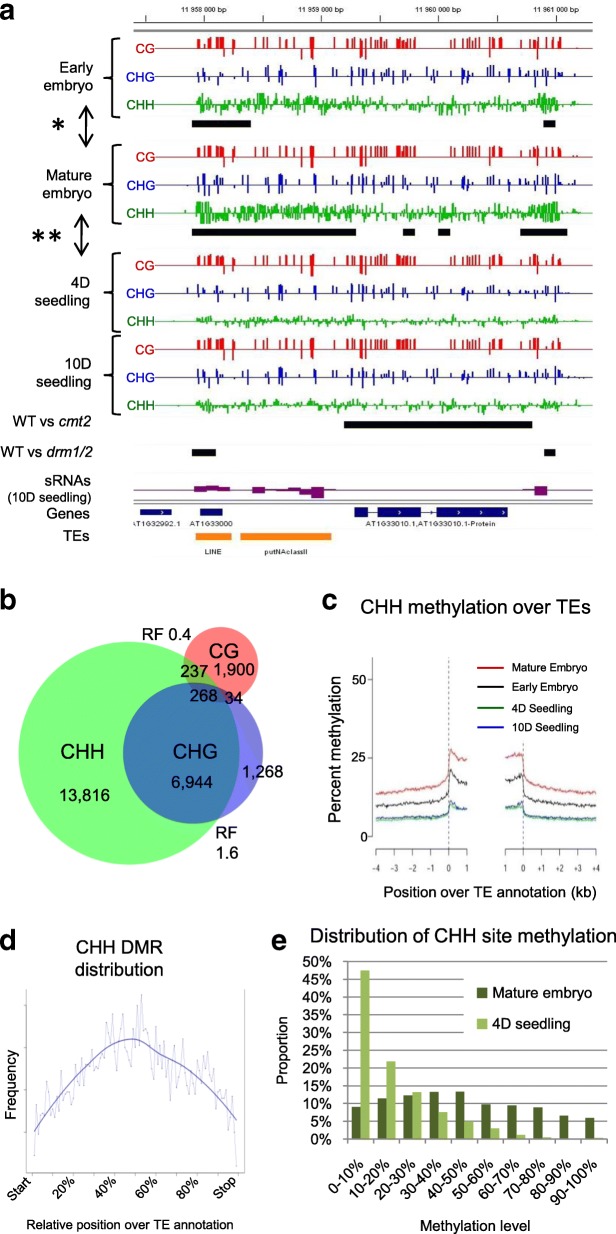
Differential methylation during early development. **a** Methylation level for each context is shown for a 3-kb window (*upper half*) of chromosome 1 for early embryo, mature embryo, four-day-old seedling, and ten-day-old seedling for each context separately (CG = *red*, CHG = *blue*, CHH = *green*). siRNA density (*violet*; ten-day-old seedlings) is indicated below. Gene models are represented in *blue* and TEs in *orange*. CHH DMRs are indicated as *black horizontal bars* for the comparisons of early vs. mature embryos (*) and mature embryos vs. four-day-old seedlings (**) as well as for WT vs. *drm1/2* (*DRM1/2*) and WT vs. *cmt2 (CMT2)* at the seedling stage [26]. **b** *Venn diagram* representing the overlap of annotated loci associated with CG, CHG, and CHH hypermethylation DMRs in the mature embryo vs. four-day-old seedling (RF = representation factor; > 1 = higher than random, < 1 = lower than random with *p* value < 1.0xe^–30^). **c** *Meta-TE plot* for all TE sequences in the range of 1–2 kb with absolute CHH methylation levels for the four developmental stages analyzed (early and late embryo, four-day-old and ten-day-old seedling). TEs of other size classes are shown in Additional file 2: Figure S8. **d** Meta-TE representation of CHH-DMR distribution of CHH hypomethylation identified between early and mature embryos over TE annotations. **e** Distribution of methylation level frequency for CHH sites in mature embryos (*dark green*) and four-day-old seedlings (*light green*) within CHH-DMRs identified between mature embryos and four-day-old seedlings

Consistent with CHG and CHH methylation levels being highest in mature embryos among the four developmental stages, CHG-DMRs and CHH-DMRs are most numerous in the pairwise comparisons involving mature embryos (Fig. 2a, b; Additional file 2: Figure S4A). CHG-DMRs and CHH-DMRs typically overlap (Fig. 2b), although CHH-DMRs tend to be larger, in agreement with the larger average size of CHH-MRs in mature embryos (Fig. 1b; Additional file 2: Figures S6A, B). The vast majority of CHG-DMRs and CHH-DMRs results from different levels of methylation of the same genomic region at the four developmental stages rather than from a de novo gain or loss of MRs (Fig. 2a; Additional file 2: Figures S6C, D; S7B). Furthermore, CHH methylation preferentially increases in the internal part of TE sequences during embryogenesis, which is typically less methylated compared to the extremities of TEs (Fig. 2c, d; Additional file 2: Figure S8) [21, 22]. In summary, around 28,300 individual CHH sites reach 100% methylation in mature embryos, a level that does not persist in seedlings (Fig. 2e; Additional file 2: Figure S9A).

### Late embryonic CHH hypermethylation matches loss of DNA methylation in the endosperm

**Fig. 3:**
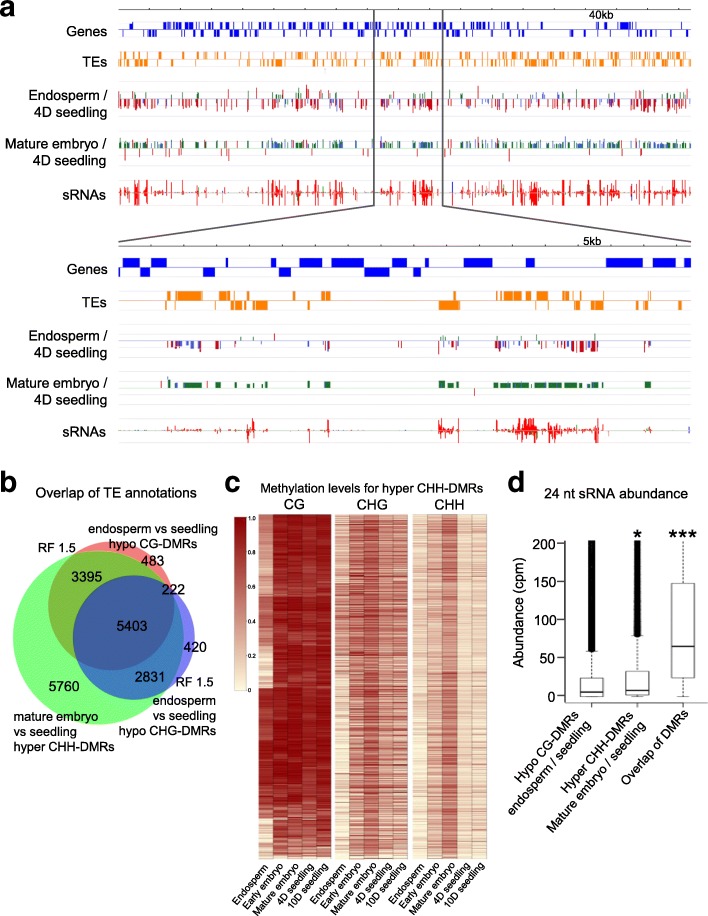
Hypermethylation in the mature embryo correspond to hypomethylation in the endosperm. **a** Representation of a 740-kb genomic region (*top*) and a zoom-in of 86 kb (*bottom*) showing genes (in *blue*) and TEs (in *orange*) as well as DMRs between endosperm [6] vs. four-day-old seedlings and mature embryos vs. four-day-old seedlings with CG = *red*, CHG = *blue*, and CHH = *green*. DNA hypomethylation is shown as *downward bars*, hypermethylation as *upward bars* in the respective comparisons. siRNA abundance for up to 50 reads is shown for 24 nt size (*red*) and 21–22 nt (*green*). **b** *Venn diagram* representing the overlap between TE annotations affected by CHH-hypermethylation DMRs in the mature embryo with CG- as well as CHG-hypomethylation DMRs in the endosperm. RF = representation factor; RF > 1 = overlap higher than random, RF < 1 = overlap lower than random, with *p* value < 1.0xe^–30^. **c** *Heat map* showing absolute values of methylation for CG, CHG, and CHH contexts at five developmental stages/tissues based on the CHH DMRs detected between mature embryos and early embryos as well as four-day-old and ten-day-old seedlings (27,528 DMRs in total). Rows were sorted by complete linkage hierarchical clustering with Manhattan distance as a distance measure using the CG methylation values and aligning the other contexts to these coordinates. **d** *Box plots* showing the distribution of seed-derived 24-nt siRNA [27] abundance over endosperm vs. four-day-old seedlings CG hypomethylation DMRs (Hypo CG-DMRs endosperm/seedling), CHH hyper-methylation DMRs in the mature embryo vs. four-day-old seedlings (Hyper CHH-DMRs embryo/seedling) and the overlap of both DMRs (Overlap of DMRs). siRNA reads of 24 nt were counted over each feature and converted to count per million. Student’s t-test in comparison of siRNA abundance of CG hypomethylation endosperm vs. four-day-old seedlings; * = *p* value < 0.05; *** = *p* value < 0.001

In plants, the maternal genome is hypomethylated in the endosperm [23]. This loss of DNA methylation that mainly affects CG and CHG sites over TE sequences is initiated in the CC by the DNA glycosylase DEMETER (DME) and correlates with the production of siRNAs, which have been proposed to move to the embryo and induce RdDM to (re)establish proper silencing of these TE sequences [10, 14, 15, 24]. This model implies that the gain of DNA methylation observed in mature embryos over a given region correlates with the loss of DNA methylation in the endosperm for the same region. To determine if this were the case, we re-analyzed previously published endosperm methylome data (6–7 days after fertilization [6]) and found that indeed CHH-hypermethylation in mature embryos tends to affect the same sequences that are hypomethylated earlier during seed development in the endosperm (Fig. 3a–c; Additional file 6: Table S5). Moreover, examination of publicly available data of seed-derived siRNAs [25] indicates that 24-nt siRNAs abundance is highest for regions that are hypomethylated in the early endosperm and gain CHH methylation in the mature embryo (Fig. 3d). This observation further supports a model whereby endosperm-derived siRNAs progressively instruct CHH methylation in the embryo.

CHH methylation in Arabidopsis is carried out by two main, mostly non-overlapping, pathways, one involving the H3K9me2-binding DNA MTase CMT2 and the other the siRNA-dependent MTases DRM1/2 [26, 27]. Indeed, fully methylated CHH sites showed distinct patterns between early and mature embryos. Specifically, CHH sites with 100% methylation in the early embryo tend to be located throughout long TE sequences, which in seedlings are preferential targeted by CMT2 (Additional file 2: Figures S9C–F; S10; Additional file 7: Table S6). In contrast, full CHH methylation in mature embryos is preferentially located at TE boundaries, a pattern typical of RdDM targets (Additional file 2: Figure S9D) [26]. In agreement, genes involved in RdDM (e.g. *AGO4*, *DMS3*, and *DRM2*) show highest expression during late embryogenesis (Additional file 2: Figure S11) [28]. Moreover, genes involved in the generation of the 24-nt siRNAs required for RdDM, such as RNA-dependent RNA polymerase 2 (*RDR2*) and *DCL3*, are found primarily in the endosperm (Additional file 2: Figure S11) [8, 28]. Taken together, these observations suggest that siRNA production in the early endosperm precedes RdDM in the embryo (Additional file 2: Figure S11).

### PRC2 activity has no direct effect on DNA methylation

**Fig. 4:**
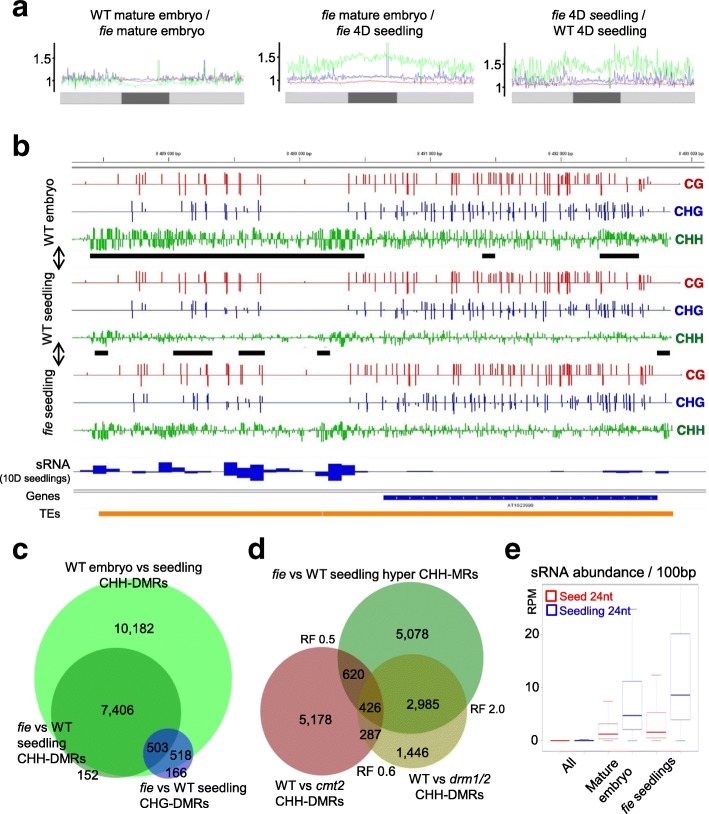
*fie* mutant seedlings partially maintain an embryonic RdDM profile. **a** Methylation ratios over 100-kb windows for CG (*red*), CHG (*blue*), and CHH (*green*) over chromosome 5 as an example in three comparisons. **b** Genome browser screenshot of a 4-kb region indicating DNA methylation levels in WT mature embryo and four-day-old seedling in WT and *fie*. Gene models are represented in *blue* and TEs in *orange* (*bottom*). CHH-DMRs are indicated as *black horizontal bars*; CG methylation is shown in *red*, CHG in *blue*, and CHH in *green*; WT seedling 24-nt siRNA (ten-day-old seedling) abundance shown as *blue bars*. **c** *Venn diagram* showing the overlap of TE annotations with CHH hypermethylation DMRs between mature embryo vs. four-day-old seedling (WT embryo vs. seedling CHH-DMRs) as well as *fie* vs. WT four-day-old seedling (*fie* vs. WT seedling CHH-/CHG-DMRs). **d** *Venn diagram* comparing *fie* vs. WT four-day-old seedlings CHH hypermethylation DMRs with DRM1/2- and CMT2-dependent CHH methylation. RF = representation factor; RF > 1 = overlap higher than random, RF < 1 = overlap lower than random, with *p* value < 1.0xe^–30^. **e** *Box plot* representing the relative abundance of 24-nt siRNAs from seeds (*red*) [25] and seedlings (*blue*) in reads per million (RPM; *y-axis*) for 100-nt windows throughout the genome (All), DMRs that show both, CG hypomethylation in the endosperm vs. four-day-old seedlings as well as CHH hypermethylation DMRs in the mature embryo vs. four-day-old seedlings (Mature embryo) and for CHH-hyper DMRs found in *fie* seedlings compared to WT seedlings (*fie* seedling)

We previously showed that viable seeds with a purely maternal endosperm can be produced by bypassing the requirement of PRC2 activity during seed development [29]. This bypass allowed us to recover homozygous *fie* mutant embryos, which have lost PRC2 activity, resulting in seedlings that lack the repressive H3K27me3 mark [30]. We took advantage of this genetic system to investigate the impact of a strictly maternal endosperm on the embryo methylome and to determine whether the absence of H3K27me3 in *fie* mature embryos affects DNA methylation [30]. This is not the case, as DNA methylation patterns are similar between WT and *fie* mature embryos, with few local, low-level variations (Fig. 4a left side; Additional file 2: Figures S6A, B; S12A–C; Additional file 8: Table S7). Thus, the absence of the paternal genome in the endosperm does not affect RdDM in the mature embryo. This result is in agreement with the observation that it is mainly the maternal genome, which is demethylated in the endosperm [6] and which would contribute to siRNA-derived methylome (re)establishment in the embryo.

DNA methylation and H3K27me3 are largely mutually exclusive and it was reported that H3K27me3 can compensate for the loss of DNA methylation, although both chromatin marks can regulate the same gene and have been found to contribute to genomic imprinting in the endosperm [31–34]. Yet, DMRs detected in the comparison between WT and *fie* mature embryos do not overlap with regions marked by H3K27me3 in WT seedlings, whether considering genes or TE sequences (Additional file 2: Figures S12D, E) [30], indicating that PRC2 activity does not interfere substantially with DNA methylation targeting.

### Elevated RdDM activity is a feature of late embryonic development

We have previously shown that PRC2 is a major regulator of the embryo to seedling transition by repressing the embryonic program after germination [30]. In comparison with four-day-old WT seedlings, *fie* seedling showed massive CHH and to a lesser extent CHG hypermethylation (Fig. 4a, b; Additional file 9: Table S8), consistent with a failure to terminate an embryonic developmental program. The majority of CHH-DMRs and CHG-DMRs found in *fie* at the seedling stage overlapped with late embryonic CHH-hypermethylated loci in the WT (Fig. 4c). Yet, in contrast to the mature embryonic methylome where CHH and CHG hypermethylation affect mainly the same TEs (85% overlap, Fig. 2d), this overlap is not as extensive in *fie* seedlings (only 42% overlap; Fig. 4c), indicating two independent pathways contributing to the elevated levels of CHH and CHG methylation in the mutant after germination. Previous transcriptional profiling of *fie* seedlings revealed increased levels of expression of RdDM genes compared to WT, including *AGO4*, *DMS3*, and *DRM2* [30], and consistently, TEs showing CHH hypermethylation in *fie* seedlings primarily depend on DRM1/2 and are largely independent of CMT2, in agreement with unaltered *CMT2* expression levels in *fie* (Fig. 4d) [30]. In agreement with this notion, 24-nt siRNAs detected in WT seedlings are highly abundant over CHH hypermethylated regions in *fie* seedlings (Fig. 4b, e) [35]. Notably, the upregulated genes involved in DNA methylation are not direct targets of PRC2 and their ectopic expression in *fie* seedlings thus primarily reflects the extension of the embryonic transcriptional program [30].

## Discussion

Our work reveals that, although not reset globally, DNA methylation is nevertheless dynamic during Arabidopsis embryogenesis and early growth. Specifically, CG methylation, which is prevalent, is likely inherited from the gametes [36, 10] and remains constantly high in the embryo as well as during vegetative growth over TE sequences. In contrast, CHG and especially CHH methylation, which is relatively low during post-embryonic development, reaches saturation at numerous sites in the mature embryo. This transient saturation is seen mostly over RdDM targets, which suggests that RdDM is active in all cells of the mature embryo. Our analysis also suggests that the CHH MTase CMT2 targets long TEs, mainly located within pericentromeric regions, early during embryogenesis. However, further work is required to determine the extent to which the CMT2-dependent and RdDM-dependent CHH methylation pathways fulfill distinct functions during embryogenesis.

The broad RdDM activity that we have uncovered in the mature embryo may reflect a need to specifically preserve every cell of the future plant from the deleterious consequences of TE activity. This is clearly not the case post-embryonically since RdDM is restricted mainly to the meristems, which contain the stem cell niches from which all plant tissues derive [37]. Although these stem cell niches are established already very early during embryogenesis, they do not contribute to a large extent to cell proliferation in the embryo, which may provide an explanation for why the entire embryo carries out RdDM, thereby assuring genome integrity in each cell.

The notion that meristematic tissues specifically maintain the embryo-derived elevated CHH methylation pattern is further supported by the finding that columella cells in the root meristem, which are separated from stem cells by one cell division only, show an elevated level of CHH methylation [38]. In agreement with the hypothesis that it is not primarily the columella cell identity but rather the proximity to the stem cells that is responsible for this effect, another columella cell population further away from the quiescent center did not show CHH hypermethylation [38]. Alternatively, it was proposed that siRNA production in columella cells serves to reinforce RdDM in the stem cell niche and maintain its homeostasis [38] and both scenarios are in agreement with elevated CHH methylation levels. An opposite situation seems to prevail in mammals, where cell stemness has been linked to DNA hypomethylation in the zygote and early embryonic development as well as during primordial germ cell differentiation, whereas high DNA methylation levels are found in somatic tissues with minor tissue-specific differences [5, 39–41]. To which extent these different epigenetic states are relevant for cell differentiation in animals and plants needs to be determined [42, 43].

In plants, cell differentiation depends to a large extent on PRC2 activity and it is noteworthy that *fie* mutants maintain high CHH methylation levels post-embryonically. It is tempting to speculate that loss of PRC2 creates a pluripotent state associated with elevated RdDM activity. However, our previous observation that *fie* mutant seedlings show de-repression of embryo-specific transcripts [30] suggests instead that the CHH methylation pattern in *fie* seedlings simply results from the persistence of an embryo-like state post germination. The role of PRC2 in controlling the passage from one developmental stage to another is also reflected in the methylome of *fie* mutant endosperm that fails to cellularize but, opposite to *fie* seedlings, shows strongly reduced CHH methylation [44]. Consistent with the assumption of developmentally regulated methylomes, WT endosperm before cellularization is devoid of CHH methylation [45]. The fact that genes involved in RdDM are not marked by H3K27me3 further indicates that RdDM is not directly controlled at the chromatin level but rather follows developmental stage- and cell-differentiation-dependent dynamics [30].

Our data suggest a gradual expansion of RdDM activity after fertilization, in agreement with previous findings based on the examination of five loci at the embryonic globular, heart, and green torpedo stage [8]. Consistent with sexual reproduction being an important determinant of CHH methylation, clonally derived plant species show strongly reduced CHH methylation levels [46]. However, the shift from clonal to sexual reproduction does not re-establish elevated CHH in a single generation, indicating that the simple passage through seed development might not be the only determinant to explain this difference [46].

It has been proposed that DNA hypomethylation in the endosperm due to the activity of the DNA glycosylase DME in the CC leads to reinforcement of RdDM in the embryo, via siRNAs [14, 15]. The mobility of siRNAs from the embryo-surrounding tissue into the embryo has not been observed directly and the endosperm tissue disintegrates during embryogenesis, leaving only a single cell-layer behind at the mature embryonic stage when CHH methylation peaks. Nonetheless, a miRNA targeting a GFP-reporter was able to silence the expression in the embryo when expressed in the CC/endosperm [6]. This suggests either that DNA hypomethylation in the early endosperm serves mainly as an initial trigger to start the process of silencing/RdDM in the embryo or that other tissues contribute to the generation of siRNAs. For instance, AGO9 is found in the integuments of early seeds and acts in a non-autonomous manner to control female gametogenesis [47]. In pollen, TE mRNAs are degraded in an AGO1/AGO2/DCL4-dependent manner to give rise to 21/22-nt siRNAs that move from the VN to the SCs [48, 49]. Indeed, PolII-dependent reactivation of TE mRNAs in the VN and endosperm may trigger the production of siRNAs of broader size range with the capacity to induce DNA methylation by a pathway that differs from PolIV-dependent RdDM, which involves mainly 24-nt siRNAs [50–52]. In line with the hypothesis that a transcription-dependent production of a methylation signal is derived from the endosperm, differential CHH methylation in the mature embryo accumulates in the inner part of TEs, not at the boarders where canonical RdDM is predominantly observed. Several lines of evidence suggest that this pathway is absent in the EC and a mutant in the PolIV subunit *NRPD1* does not show a reduction of CHH methylation in the EC [12]. Based on these observations, we propose that either non-canonical RdDM takes place in the EC and possibly also in the embryo and/or siRNA production and the consequential DNA methylation are spatially uncoupled. In support of the latter possibility, it has been shown that intercellular mobile siRNAs mediate DNA methylation outside their source of production [53, 54]. Yet, the extent to which mobile siRNAs influence embryonic CHH methylation remains to be determined.

## Conclusion

Here we have shown that establishment of CHH methylation over TE sequences is a developmentally regulated process with an unexpected increase affecting most if not all cells in mature embryos (see graphical model in Additional file 2: Figure S13). We also provide evidence that embryonic CHH methylation occurs through two pathways, H3K9me2-dependent CMT2 and RdDM, which have distinct targets and act early and late during embryogenesis, respectively. Moreover, our findings indicate that DNA hypomethylation in the endosperm likely directs CHH hypermethylation in the embryo, possibly via mobile siRNAs. However, methylation patterns in the embryo and the endosperm appear temporarily shifted with respect to each other as RdDM peaks in late embryogenesis yet affects loci that show DNA hypomethylation early during endosperm formation. Finally, the observation that fully methylated CHHs are only seen transiently, in the mature embryo, implies that the proportion of cells that carry out RdDM after germination is reduced, consistent with RdDM being mainly restricted to meristematic tissues.

## Methods

### Plant material and growth conditions

WT Col-0 and *fie* mutants [30] were grown on ½ MS plates containing 1% sucrose following a 16 h light/23 °C – 8 h dark/18 °C cycle. WT and *fie* (*AT3G20740*, SALK-T-DNA allele SALK_042962 described in [30]) plants were collected four days after germination and DNA extracted using PureLink DNA purification kit (Life Technologies). Several aliquots of Col-O WT seeds were hand-dissected and collected in liquid ½ MS solution, washed with H_2_O bidest four times, liquid removed, and frozen at – 80 °C. Embryonic *fie* mutants were selected due to their enhanced dormancy, hence non-germinating seeds seven days after germination-induction were separated and hand-dissected similarly to the WT and genotyped for the *fie* locus (see “Oligosequences”) to ensure homozygousity of the mutant embryos. Around 2000 mature embryos were used and homogenized with metallic beads using TissueLyser (Qiagen) followed by DNA extraction (PureLink DNA purification kit, Life Technologies). DNA purity and quality were controlled using Qubit fluometry and agarose gel electrophoresis. Between 1 and 2 ug of genomic DNA were bisulfite treated (EZ DNA Methylation-Gold kit, Zymo) and pair-end sequenced using BGI Tech Solutions WGBS service/Illumina 2000 sequencing technology. Sequencing data are deposited in GEO with accession GSE85975.

### Bioinformatic analysis

TAIR10 gene and TE models were obtained from The Arabidopsis Information Resource (www.arabidopsis.org). Bisulfite converted sequencing reads were mapped to reference genomes (Release 10) using the Bowtie2 alignment algorithm allowing one mismatch and only uniquely mapped reads were used for further analysis [55]. DMRs were defined by comparing the methylation level of 100-bp windows throughout the genome between two genotypes using the methylkit pipeline [56]. Bisulfite-Seq Analysis DMRs were defined by tiling the genome into 100-bp bins and comparing the number of called Cs and Ts in mutant and WT. Bins with absolute methylation difference of 0.4, 0.2, 0.2 for CG, CHG, CHH, respectively, and Benjamini–Hochberg corrected false discovery rate < 0.01 (Fisher’s exact test) were selected. To avoid 100-bp bins with few cytosines, we selected bins with at least four cytosines that are each covered by at least ten reads and maximum 100 reads in each sample. Only regions called significant in all three comparisons were defined as DMRs. Finally, because loss and gain of methylation occurred in clusters, DMRs within 100 bp of each other were merged by allowing a gap of one window.

For the siRNA comparison with DMRs, 21–22-nt and 24-nt reads were selected before mapping against the reference genomes (Release 10) using the Bowtie alignment algorithm allowing one mismatch. Then, reads were assigned to the DMRs using bedtools [58], a normalization step was done by dividing the number of reads covering the DMRs with the total number of library sRNAs that was mapping against the reference genome.

### Comparative analysis

Visualization of DNA methylation was carried out using IGV software (Broad institute). DMR comparison was illustrated using SignalMap genome viewer (Roche). Venn diagrams were generated using BioVenn web application ([57] http://www.cmbi.ru.nl/cdd/biovenn/) and statistical significance tests applied (http://nemates.org/MA/progs/overlap_stats.html). Expression analysis was carried out using Genevestigator software (Nebion) and publicly available data [58]. For tissue-specific expression visualization, we made use of the online EFP-browser (www.bar.utoronto.ca/efp/cgi-bin/efpWeb.cgi), based on published expression data [59, 60]. Global DNA methylation was compared to published data on WGBS of﻿ wild type leaf tissue [61].

### Meta-TE analysis

TAIR10 annotated TEs were grouped into three categories based on their length. For each category, TE ends were aligned and average methylation levels were plotted within 10-nt intervals. The intervals cover the inside of the TEs starting from the boarders toward the middle and 4 kb away from the TEs. To ensure common and non-overlapping intervals between annotation units belonging to the same category, the distance covered by intervals inside each unit was fixed and selected such as the shortest unit could be fully covered. For relative distributions over TEs of 100% methylated CHH and CHH hypermethylated DMRs identified between mature and early embryos, relative positions of the 100% methylated CHH and midpoints of CHH hypermethylated DMRs were computed over corresponding TEs and the sum of occurrences per relative position was plotted.

### Heat-map visualization

Heat maps show the methylation level within DMRs (rows) across all developmental stages (columns). Methylation levels within a DMR were defined as the average methylation levels of all covered cytosines that overlap the DMR. Cytosines with no coverage in any developmental stage were discarded from the analysis. DMRs covered in all developmental stages (32 DMRs discarded out of 27,560 CHH DMRs) were selected and average methylation levels for each context within CHH-DMR were computed and shown as a heat map where rows were sorted by complete linkage hierarchical clustering with Manhattan distance as a distance measure using the CG-methylation level.

#### Oligosequences

**Table:** 

Genotyping primer	Annotation	Sequence
FIE042-LP	AT3G20740	ATGTTTCACTGAGGCCATTTG
FIE042-RP	AT3G20740	ACAGGATCTCGTTGTCCACAC
SALK-LB3.1	SALK T-DNA	ATTTTGCCGATTTCGGAAC
TG9_LP	AT3G48750	TCAAACAAGTTTGGTTTTGGC
TG9_RP	AT3G48750	TTCCTTGTTCATATGTTCCCG

## Additional files

Additional file 1: Table S1. Global data analysis of bisulfite converted whole genome sequencing data. Mapping quality, deduplication and conversion rate are indicated as well as cytosines detected as methylated or unmethylated for each context. (XLSX 10 kb)

Additional file 2: Figures S1 - S13. **Figure S1.** Global methylome evaluation during four developmental stages in the WT and two developmental stages in the *fie*. **Figure S2.** Global DNA methylation ratios between developmental stages over chromosomes 2–5 using 100-kb windows. **Figure S3.** Genome-wide correlation between methylated windows as density plots along the entire genome. **Figure S4.** Characterization of DMRs. Number and distribution of annotations affected by DMRs for each context during developmental progression. **Figure S5.** Analysis of CG DMRs. **Figure S6.** Quantitative characterization of CHH-DMRs and CHG-DMRs. **Figure S7.** Global comparison of MRs for different stages and genotypes. Number of MRs at different developmental stages for each context and its distribution over annotation units. **Figure S8.** Characterization of CHH DMRs. TE-sizes dependent meta-TE plots for CHH methylation at different developmental stages. **Figure S9.** Characterization of CHH sites reaching full methylation. **Figure S10.** TE families affected by differential CHH methylation. Distribution of TE families showing CHH hypermethylation in mature embryos and *fie*seedlings as well as CMT2- and DRM1/2-dependent CHH methylated TE families. **Figure S11.** Expression patterns of genes involved in RdDM during development. Schematic heat-map representation of relative abundance of transcripts in different compartments during seed development and seedlings. **Figure S12.** Global analysis of DNA methylation in *fie*vs. WT. Level and length of methylated regions in *fie* compared to WT mature embryos and 4-day old seedlings as well as their overlap with annotations showing H3K27me3 in WT seedlings. ** Figure S13.** Model describing DNA methylation dynamics during embryogenesis. Schematic model summarizing our results and suggesting establishment of CHH methylation by distinct pathways during embryogenesis, later on maintained in the meristems after germination. (PDF 1352 kb)

Additional file 3: Table S2. DMRs detected between early and mature embryos. DNA hyper- and hypomethylated regions in the comparison between WT early embryos (raw data taken from reference [11]) and mature embryos for genes and TEs and each context are given, indicating Start and Stop of the region and its association with an annotation unit (TAIR10). (XLSX 2442 kb)

Additional file 4: Table S3. DMRs detected between mature embryos and four-day-old seedlings. DNA hyper- and hypomethylated regions identified in the comparison between WT mature embryos and four-day-old seedlings for genes and TEs and each context are given, indicating Start and Stop of the region and its association with an annotation unit (TAIR10). (XLSX 3838 kb)

Additional file 5: Table S4. DMRs detected between four-day-old and ten-day-old seedlings. DNA hyper- and hypomethylated regions identified in the comparison between WT four-day-old and ten-day-old seedlings for each context are given, indicating Start and Stop of the region and its association with an annotation unit (TAIR10). (XLSX 1104 kb)

Additional file 6: Table S5. DMRs detected between endosperm and four-day-old seedlings. DNA hyper- and hypomethylated regions in the comparison between WT endosperm (raw data taken from [11]) and mature embryos for genes and TEs and each context are given, indicating Start and Stop of the region and its association with an annotation unit (TAIR10). (XLSX 1562 kb)

Additional file 7: Table S6. CHH sites showing 100% methylation. Genome position, read coverage, and sequence of fully methylated CHH sites detected in early embryos (raw data taken from reference [11]), mature embryos, four-day-old, and ten-day-old seedlings as well as TEs associated with fully methylated CHH sites in early and mature embryos. (XLSX 1135 kb)

Additional file 8: Table S7. DMRs detected between *fie* and WT mature embryos. DNA hyper- and hypomethylated regions in the comparison between *fie* and WT mature embryos for genes and TEs and each context are given, indicating Start and Stop of the region and its association with an annotation unit (TAIR10). (XLSX 934 kb)

Additional file 9: Table S8. DMRs detected between *fie* and WT four-day-old seedlings. DNA hyper- and hypomethylated regions in the comparison between *fie* and WT four-day-old seedlings for genes and TEs and each context are given, indicating Start and Stop of the region and its association with an annotation unit (TAIR10). (XLSX 1167 kb)
