# Supplementary material for: DNA methylation dynamics during early plant life
Source: Genome Biol. 2017 Sep 25;18:179. doi: 10.1186/s13059-017-1313-0 (PMC5611644; doi:10.1186/s13059-017-1313-0)
Supplement: Supplementary file 2 — Figure S1. Global methylome evaluation during four developmental stages in the WT and two developmental stages in the fie. Figure S2. Global DNA methylation ratios between developmental stages over chromosomes 2–5 using 100-kb windows. Figure S3. Genome-wide correlation between methylated windows as density plots along the entire genome. Figure S4. Characterization of DMRs. Number and distribution of annotations affected by DMRs for each context during developmental progression. Figure S5. Analysis of CG DMRs. Figure S6. Quantitative characterization of CHH-DMRs and CHG-DMRs. Figure S7. Global comparison of MRs for different stages and genotypes. Number of MRs at different developmental stages for each context and its distribution over annotation units. Figure S8. Characterization of CHH DMRs. TE-sizes dependent meta-TE plots for CHH methylation at different developmental stages. Figure S9. Characterization of CHH sites reaching full methylation. Figure S10. TE families affected by differential CHH methylation. Distribution of TE families showing CHH hypermethylation in mature embryos and fieseedlings as well as CMT2- and DRM1/2-dependent CHH methylated TE families. Figure S11. Expression patterns of genes involved in RdDM during development. Schematic heat-map representation of relative abundance of transcripts in different compartments during seed development and seedlings. Figure S12. Global analysis of DNA methylation in fievs. WT. Level and length of methylated regions in fie compared to WT mature embryos and 4-day old seedlings as well as their overlap with annotations showing H3K27me3 in WT seedlings. Figure S13. Model describing DNA methylation dynamics during embryogenesis. Schematic model summarizing our results and suggesting establishment of CHH methylation by distinct pathways during embryogenesis, later on maintained in the meristems after germination. (PDF 1352 kb) [file 13059_2017_1313_MOESM2_ESM.pdf]

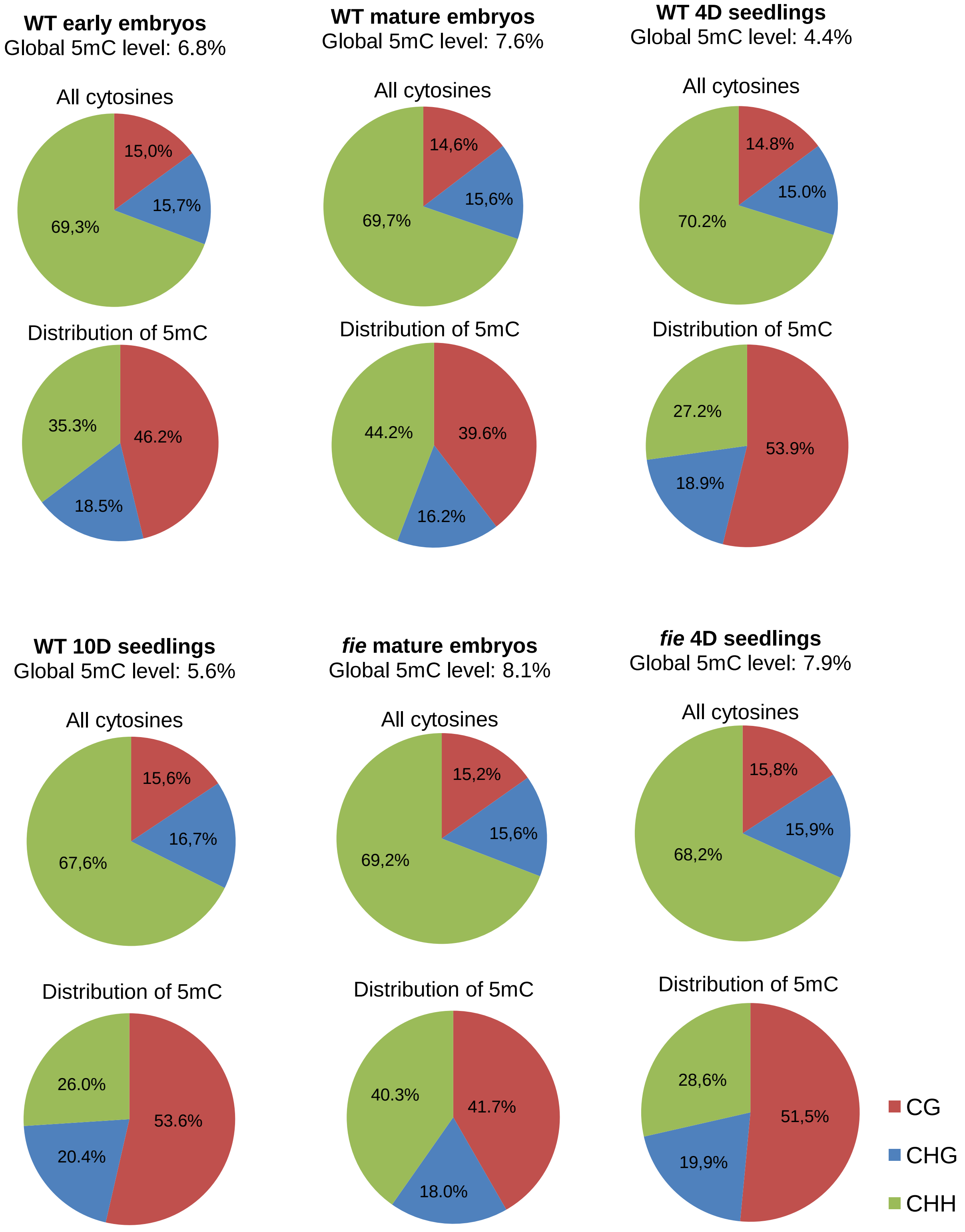

Figure S1. Global methylome evaluation during 4 developmental stages in wild-type and 2 developmental stages in the *fie* mutant

For all samples used in this study the entire cytosine- and methylated cytosine reads in their respective context are shown (Col-0 wild type early embryo [11], mature embryo, 4- and 10-day-old seedling as well as *fie* mature embryo and 4-day-old seedling).

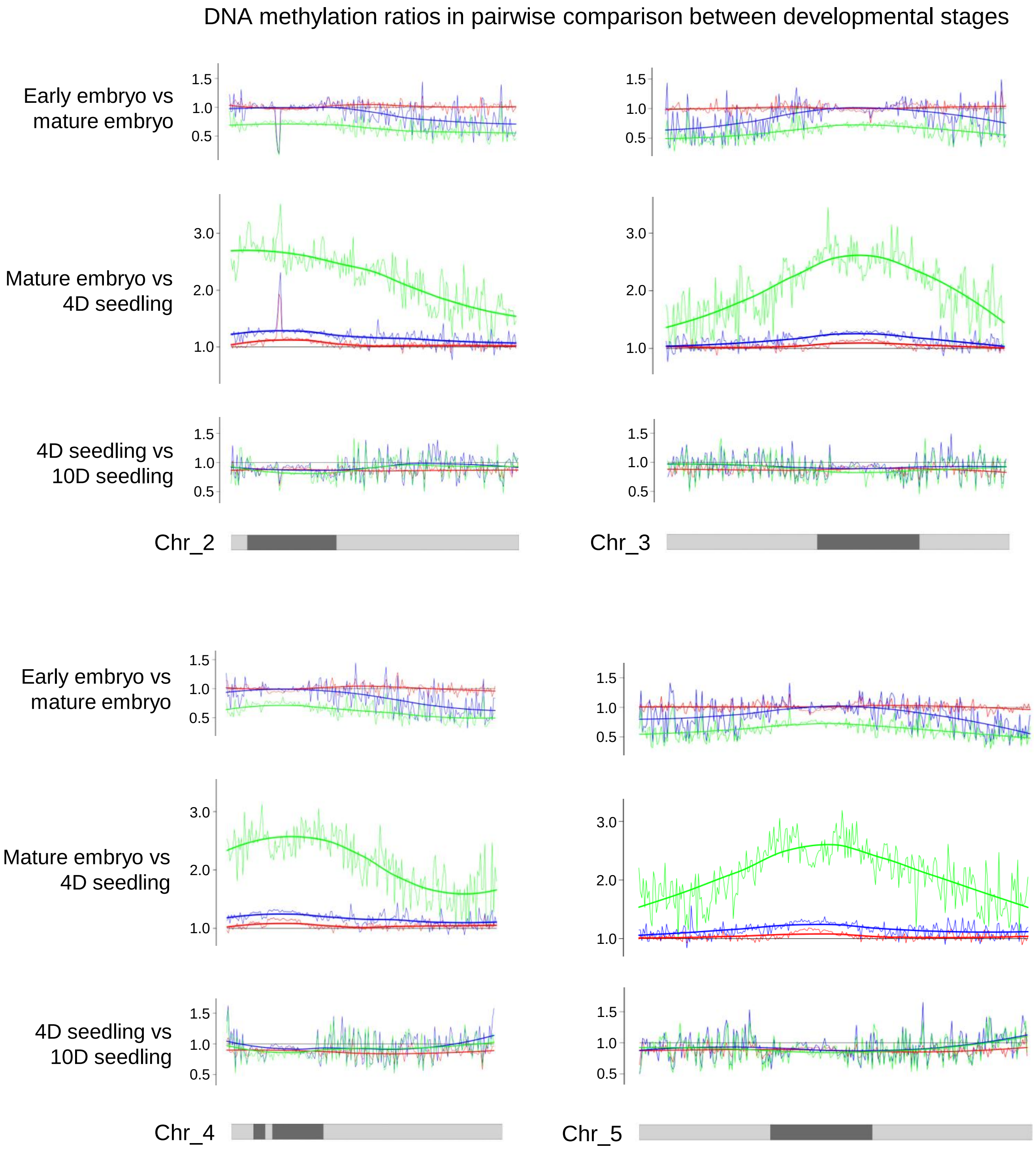

Figure S2. Global DNA methylation differences during developmental progression
Methylation ratios of 100 kb windows for CG (red), CHG (blue) and CHH (green), represented as linear regression curve over chromosomes 2-5 as described in Figure 1C-E for chromosome 1.

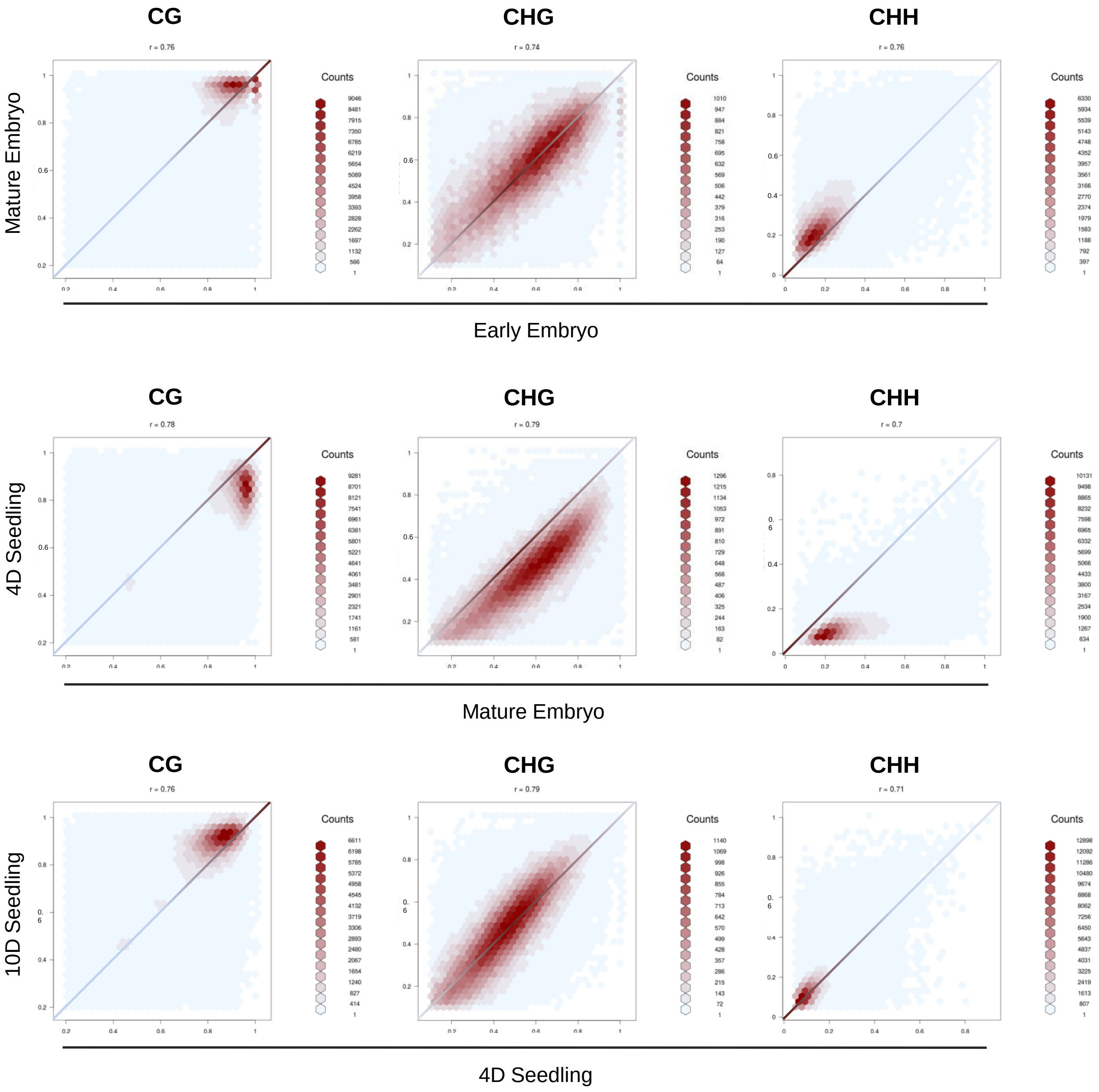

Figure S3. Genome-wide correlation between methylated windows

Density plots correlating DNA methylation levels for each context along the entire genome over 100nt windows. Only values with a coverage between 10 and 100 reads and a minimum percentage of 20%, 10% and 5% methylation for CG, CHG and CHH, respectively, are taken into account.

A

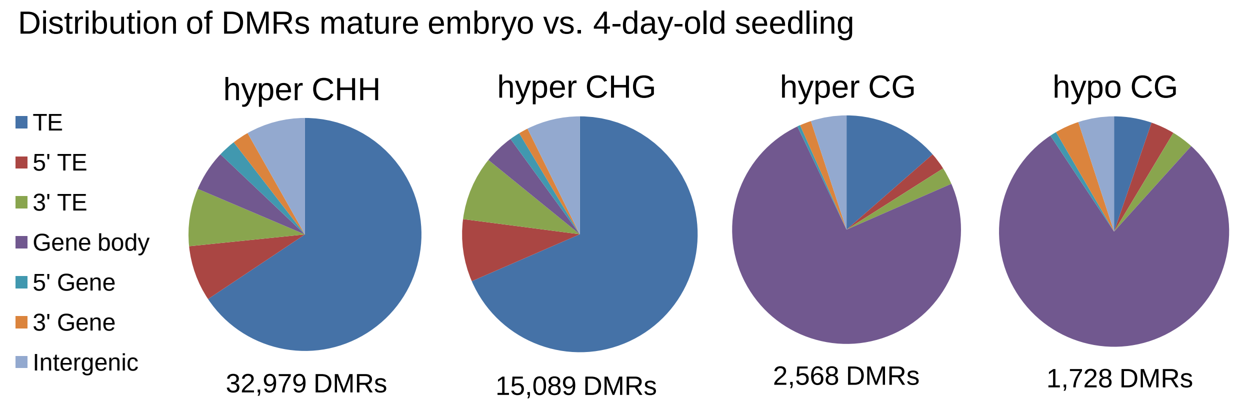

B

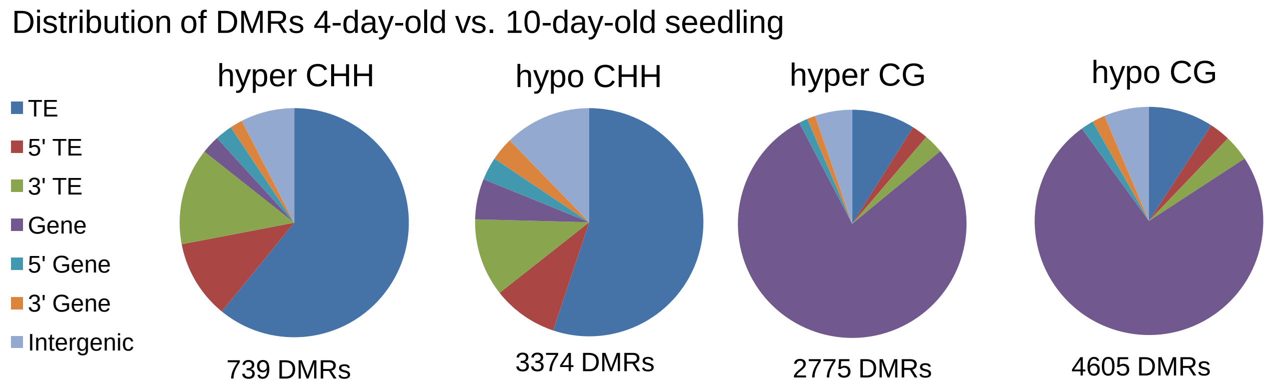

Figure S4. Characterization of DMRs

(A) Number and distribution of DMRs detected between mature embryos and 4-day-old seedlings with respect to the underlying annotation or intergenic region for CHH hypermethylation DMRs, CHG hypermethylation DMRs and CG hyper- and hypomethylation DMRs. CHH and CHG hypomethylation DMRs are almost absent in this comparison and not shown.

(B) Number and distribution of DMRs detected between 4- and 10-day-old seedlings with respect to the underlying annotation or intergenic region for CHH and CG hyper- and hypomethylation DMRs (CHG DMRs are very limited in number and not shown here).

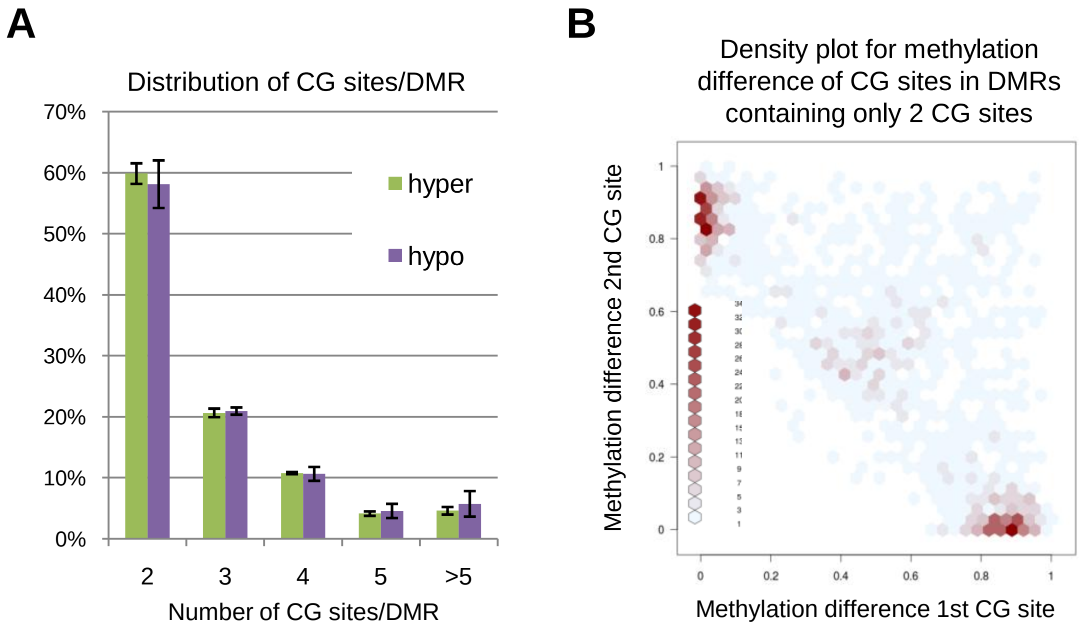

Figure S5. Analysis of CG DMRs

(A) Distribution of CG sites within hypermethylated and hypomethylated CG-DMRs, for the four comparisons (early vs. mature embryo, mature embryo vs. 4-day-old seedling, 4-day- vs. 10-day-old seedling).

(B) Density plot of the methylation differences for the two CG sites of CG-DMRs that contain only two such sites. In most of the cases, only one of the two CG sites is differentially methylated.

Figure S6. Quantitative characterization of CHH- and CHG- DMRs (next side)

(A) DMR size distribution of CHH- (green) and CHG- (blue) DMRs.

(B) Distribution of methylation level differences at CHH- (green) and CHG- (blue) DMRs.

(C) Overlap of hypermethylated CHH-DMRs detected between early and mature embryo with hypomethylated CHH-DMRs detected between mature embryo and 4-day-old (4D) or 10-day-old (10D) seedling.

(D) Overlap of CHG-MRs detected at any of the four developmental stages and hypermethylated CHG-DMRs detected between mature embryo and 4-day-old seedlings as well as hyper- and hypomethylated CHG-DMRs detected between early and mature embryo.

(E) Screen shot showing size and level of CHH-DMRs identified in different comparisons (wild type mature embryos vs. 4-day-old seedlings; wild type vs. *fie* mature embryos; wild type vs. *fie* 4-day-old seedlings). Overlap of CHH-DMRs between comparisons is shown as light green vertical bars. For each comparison, hypermethylation is shown as upward bars, hypomethylation as downward bars. Gene models are represented in blue, TEs in orange and sRNA abundance in red (24nt) or green (21-22nt).

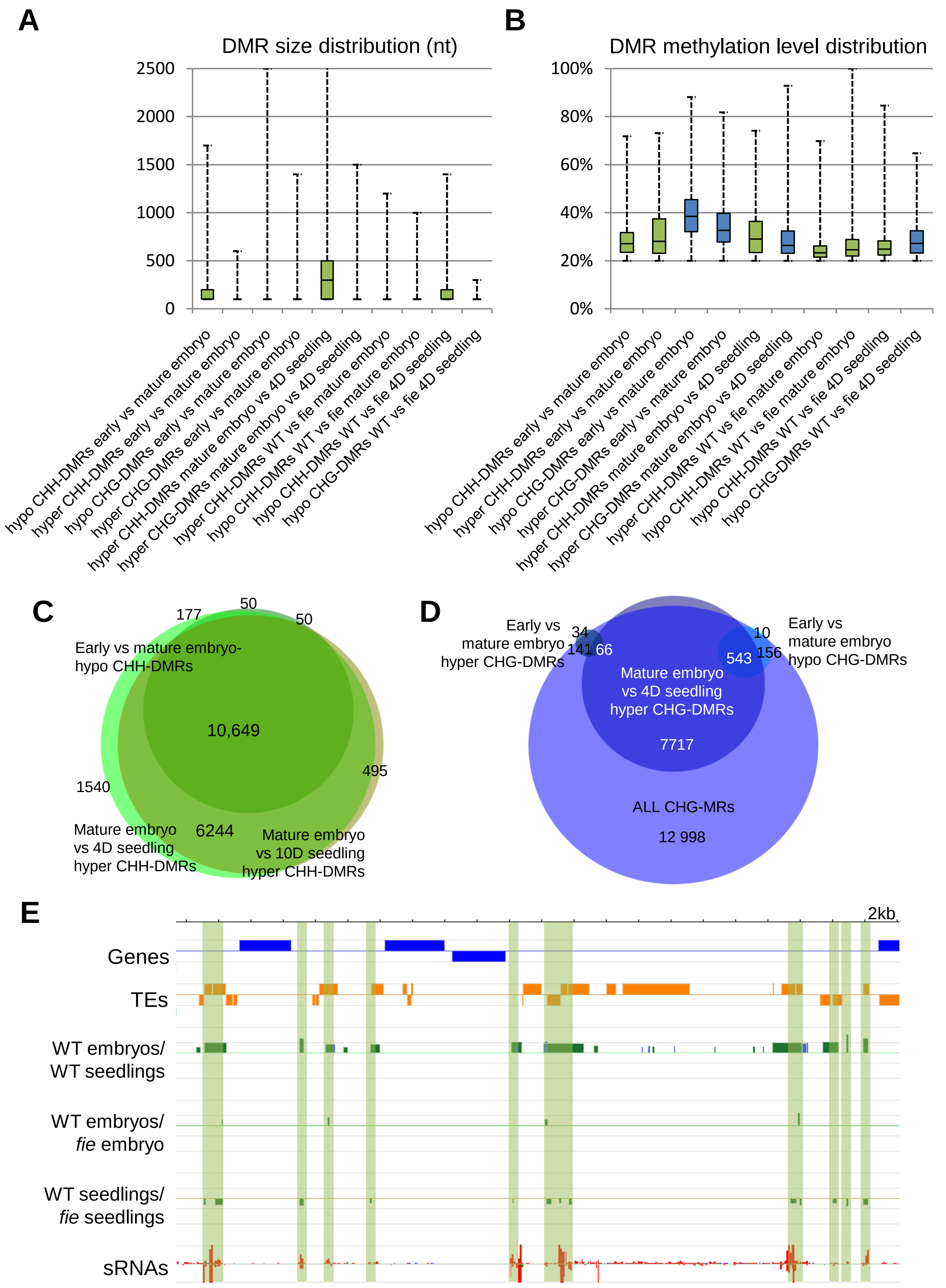
A
DMR size distribution (nt)
2500
2000
1500
1000
500
0
hypo CHH-DMRs early vs mature embryo
hyper CHH-DMRs early vs mature embryo
hypo CHG-DMRs early vs mature embryo
hyper CHG-DMRs early vs mature embryo
hyper CHH-DMRs mature embryo vs 4D seedling
hyper CHG-DMRs mature embryo vs 4D seedling
hyper CHH-DMRs WT vs fie mature embryo
hypo CHH-DMRs WT vs fie mature embryo
hypo CHH-DMRs WT vs fie 4D seedling
hypo CHG-DMRs WT vs fie 4D seedling
B
DMR methylation level distribution
100%
80%
60%
40%
20%
0%
hypo CHH-DMRs early vs mature embryo
hyper CHH-DMRs early vs mature embryo
hypo CHG-DMRs early vs mature embryo
hyper CHG-DMRs early vs mature embryo
hyper CHH-DMRs mature embryo vs 4D seedling
hyper CHG-DMRs mature embryo vs 4D seedling
hyper CHH-DMRs WT vs fie mature embryo
hypo CHH-DMRs WT vs fie mature embryo
hypo CHH-DMRs WT vs fie 4D seedling
hypo CHG-DMRs WT vs fie 4D seedling
C
177
50
50
Early vs mature embryo-hypo CHH-DMRs
10,649
495
1540
Mature embryo vs 4D seedling hyper CHH-DMRs
6244
Mature embryo vs 10D seedling hyper CHH-DMRs
D
Early vs mature embryo hyper CHG-DMRs
34
141
66
10
543
156
Early vs mature embryo hypo CHG-DMRs
Mature embryo vs 4D seedling hyper CHG-DMRs
7717
ALL CHG-MRs
12 998
E
2kb
Genes
TEs
WT embryos/ WT seedlings
WT embryos/ fie embryo
WT seedlings/ fie seedlings
sRNAs

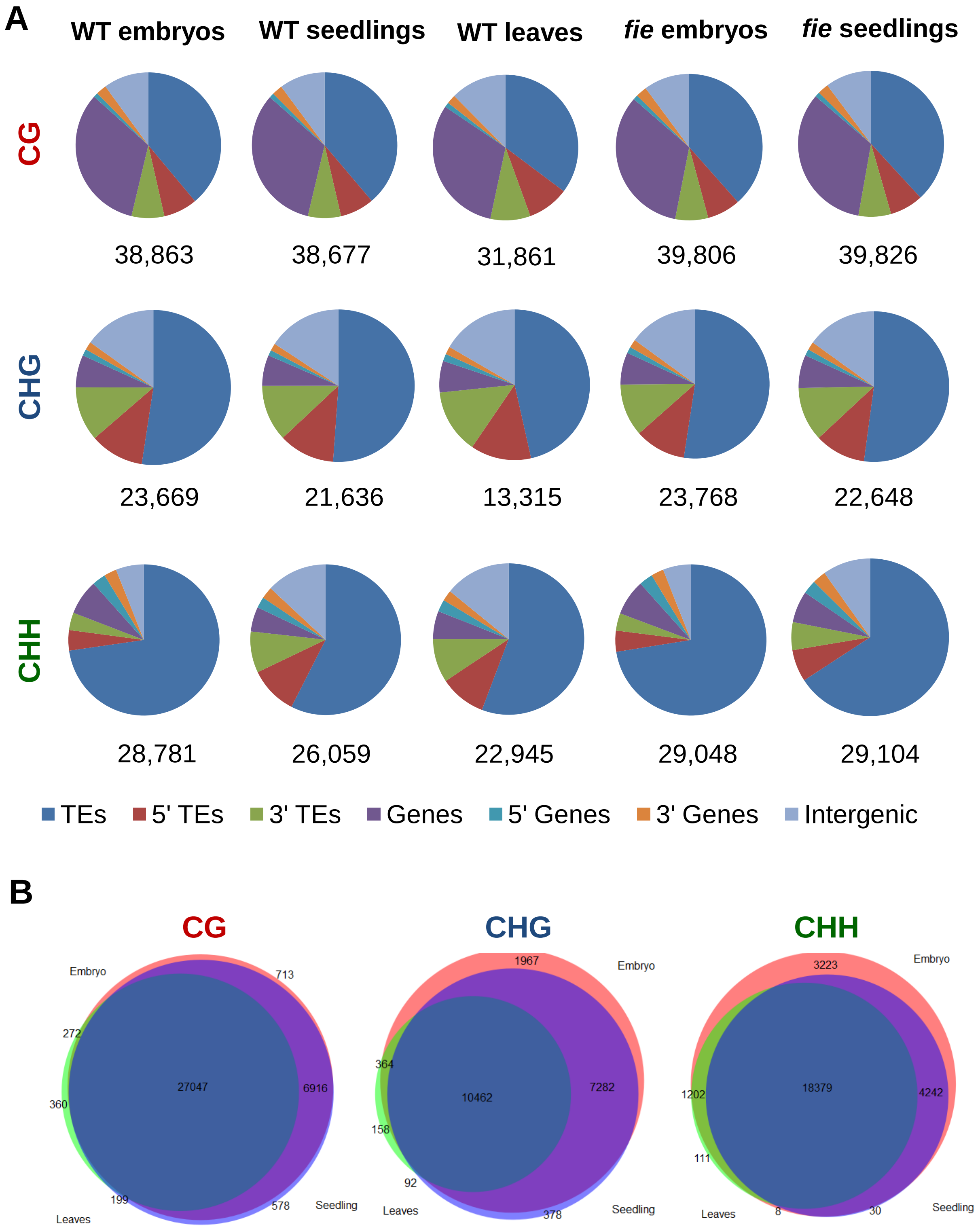

Figure S7. Global comparison of MRs for different stages and genotypes

(A) Distribution of MRs for CG, CHG and CHH context with respect to annotated domains (Genes or TEs and their 5' and 3' regions) or intergenic regions for 4 samples generated in this study (mature embryos and 4-day-old seedling for wild type [WT] and *fie* and from leave material (WT leaves) published previously [61].

(B) Overlap of MR-associated annotations between mature embryos (Embryo), 4-day-old seedlings (Seedling) and leaves from mature plants (Leaves; from reference [61]).

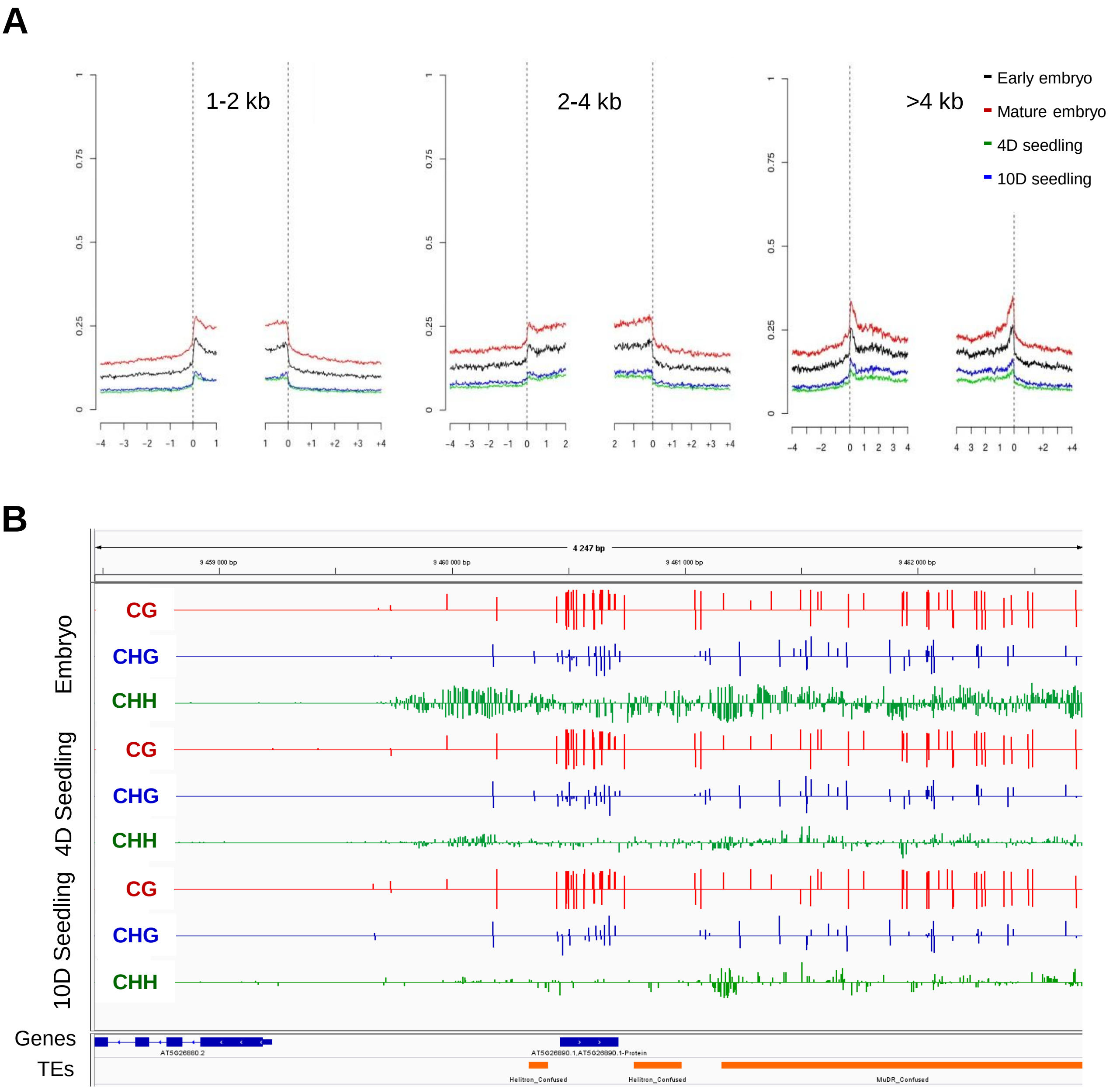

Figure S8. Characterization of CHH DMRs

(A) Meta-TE representation for TEs showing CHH DMRs with absolute CHH methylation values with 4kb up- and downstream of the annotation unit for all 4 developmental stages analyzed (early and mature embryo, 4- and 10-day-old seedling) and separated into different sequence lengths.

(B) Examples of genomic regions showing globally high CHH methylation in mature embryos (Embryo) covering entire annotated domains in comparison with 4- (4D seedling) and 10-day-old seedlings (10D seedling) where CHH methylation is globally lost after germination and maintained mainly at the flanking regions of the annotated domains. CHH in green, CHG in blue, CG in red; genes in blue, TEs in orange. Methylated gene AT5G26890 is a hypothetical gene/codes for an unknown protein.

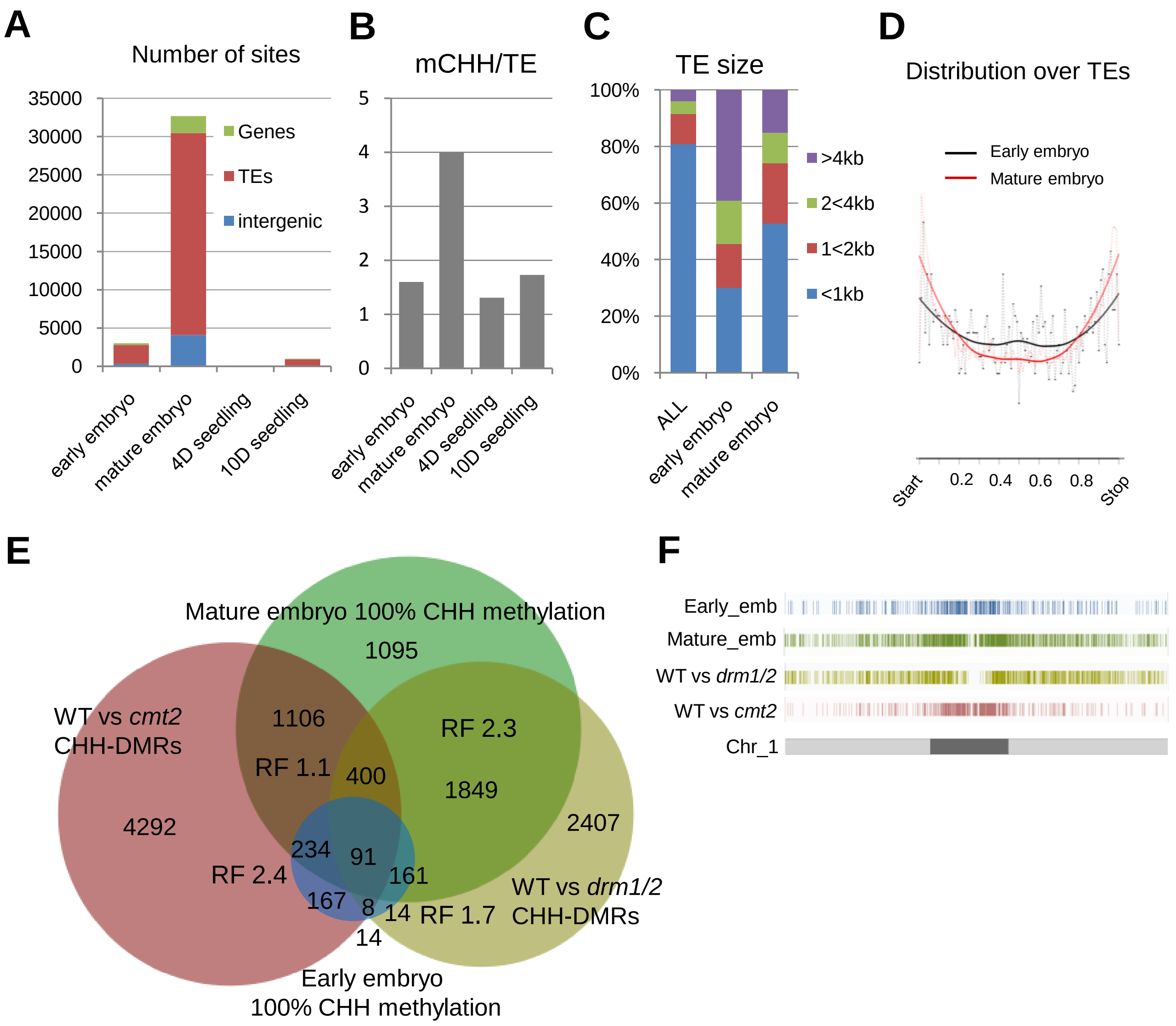

Figure S9. Characterization of CHH sites reaching full methylation

(A) Distribution and number of saturating methylation of CHH sites in early development over genes, TEs and intergenic regions of early and mature embryos as well as 4- and 10-day-old seedlings.

(B) Fully methylated CHH sites per TE at different stages.

(C) Size of TEs affected by saturating CHH methylation in early and mature embryos compared to size distribution of all TEs.

(D) Meta-TE analysis showing the relative distribution of 100% methylated CHH sites along the affected TE annotation for early (red line) and mature (black line) embryos.

(E) Venn diagram showing the overlap of 100% methylated CHH sites in early and mature embryos with DRM1/2- and CMT2-dependent CHH methylation [26]. RF=representation factor; RF>1 = overlap higher than random, RF<1 = overlap lower than random, with p-value <$1.0xe^{-30}$.

(F) Distribution of CHH sites showing 100% methylation along chromosome 1 for early and mature embryos, respectively as well as CMT2 and DRM1/2-dependent CHH methylation. Pericentromeric region is shown as dark grey.

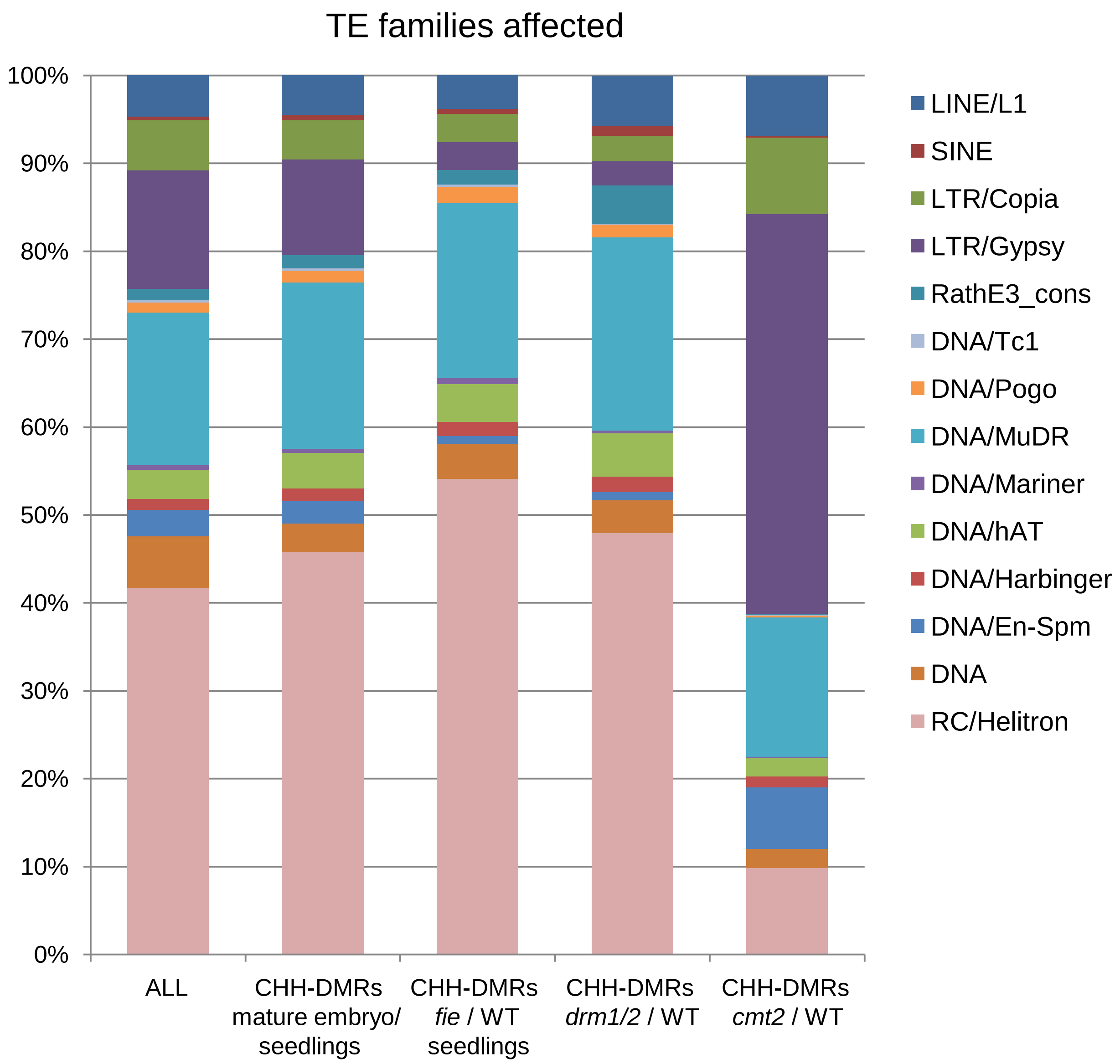

Figure S10. TE families affected by differential CHH methylation

Representation of the relative distribution of TE families within the genome (ALL) and those showing differential CHH methylation in the comparison of mature embryos and 4-day-old seedlings, *fie* vs wild type 4-day-old seedlings, *cmt2* vs. wild type (CMT2) and *drm1/2* vs. wild type (DRM1/2).

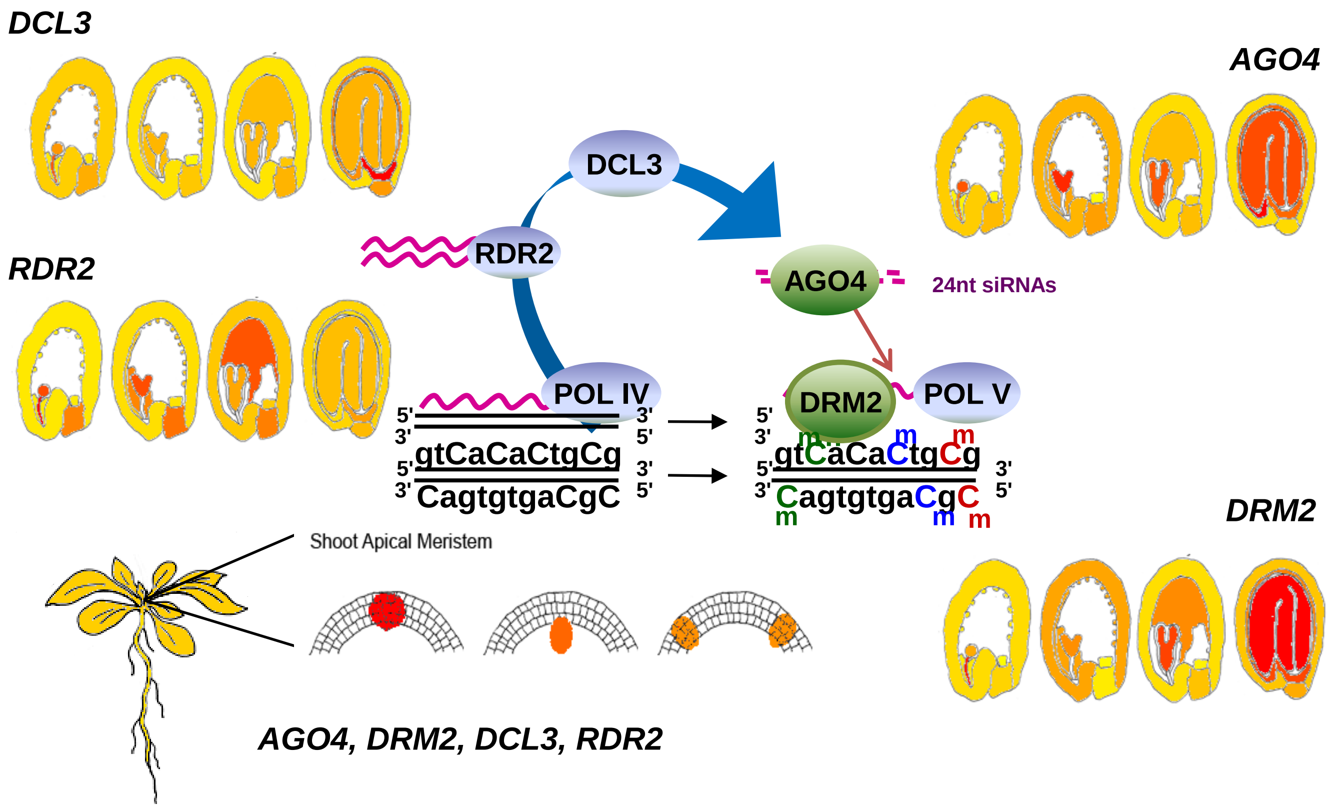

Figure S11. Expression patterns of genes involved in RdDM during development
Simplified model representing the members of the canonical RdDM pathway that relies on the production of PolIV-derived non-coding transcripts that give rise to double stranded RNA by RDR2, which are processed into 24nt siRNAs by DCL3. These siRNAs are incorporated into AGO4, which guides DRM2 to methylate the siRNA-matching target loci. Components encircled in green represent genes ectopically expressed in *fie* [30]. Four stages of seed development with tissue-specific expression patterns are shown [28], together with a schematic representation of the RdDM members with low global expression level in the vegetative tissue but elevated levels in the meristem [59]. Graphical tissue-specific representation was generated using the Arabidopsis eFP-browser with absolute expression values and their relative distribution between different tissues as setting (http://www.bar.utoronto.ca/efp).

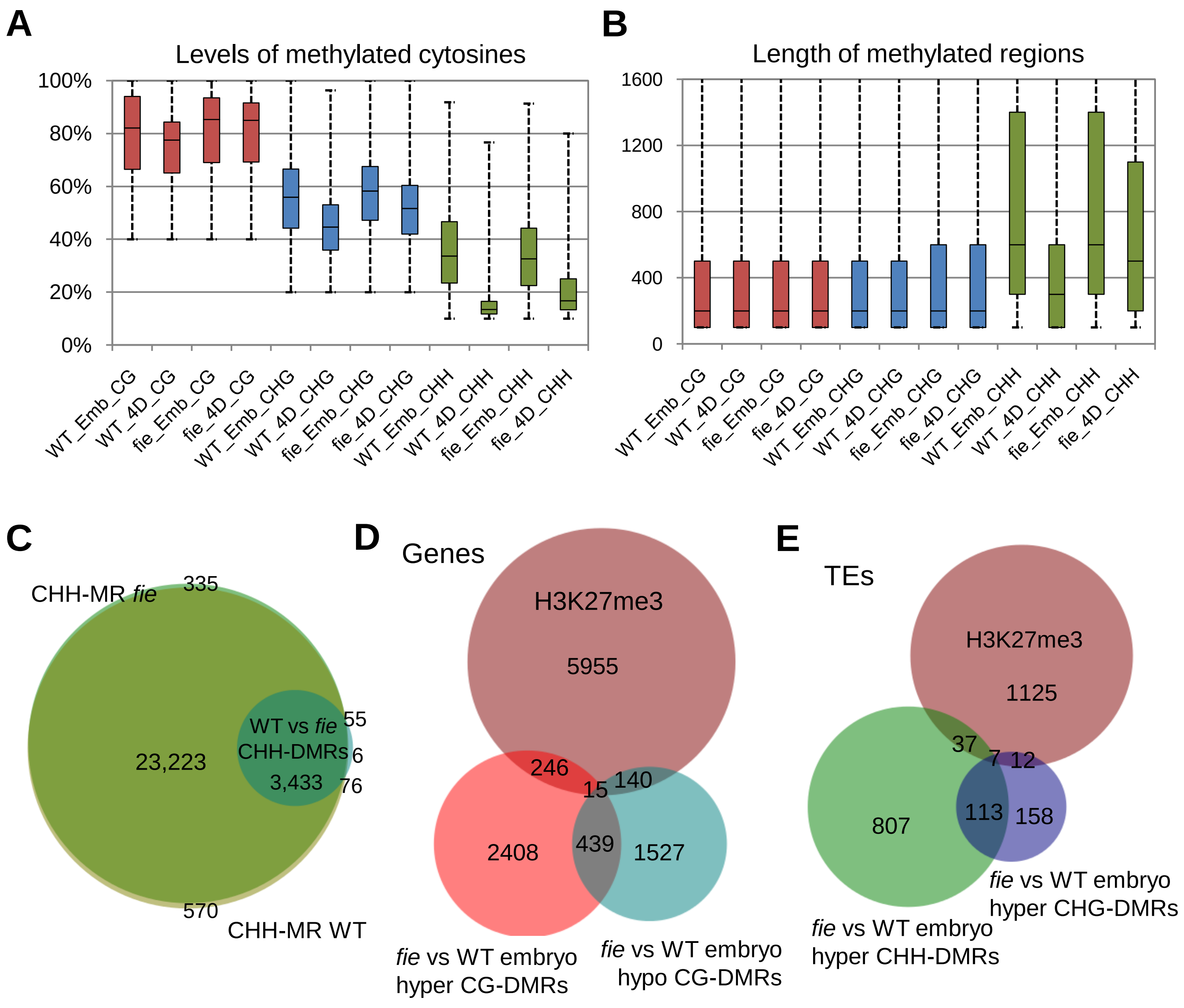

Figure S12. Global analysis of DNA methylation in *fie* vs. wild type

(A)-(B) Box plot representing the distribution of DNA methylation levels (A) and length of MRs (B) of mature embryos and 4-day-old seedlings in wild type and *fie*, respectively for each context.

(C) Venn diagram representing the overlap of annotations showing CHH-MRs in wild type and *fie* mature embryo as well as CHH DMRs (hyper and hypo) between *fie* and wild type mature embryos.

(D) Venn diagram showing the overlap of genes with elevated (CG hyper DMRs *fie* vs WT embryo) or reduced (CG hypo DMRs *fie* vs WT embryo) CG methylation in *fie* compared to wild type mature embryos with H3K27me3-marked loci in seedlings [30].

(E) Venn diagram showing overlap of TEs marked by H3K27me3 [30] and TEs gaining CHH or CHG methylation in *fie* vs. wild type mature embryos. RF=representation factor; RF>1 = overlap higher than random, RF<1 = overlap lower than random, with p-value <1.0xe^{-30}.

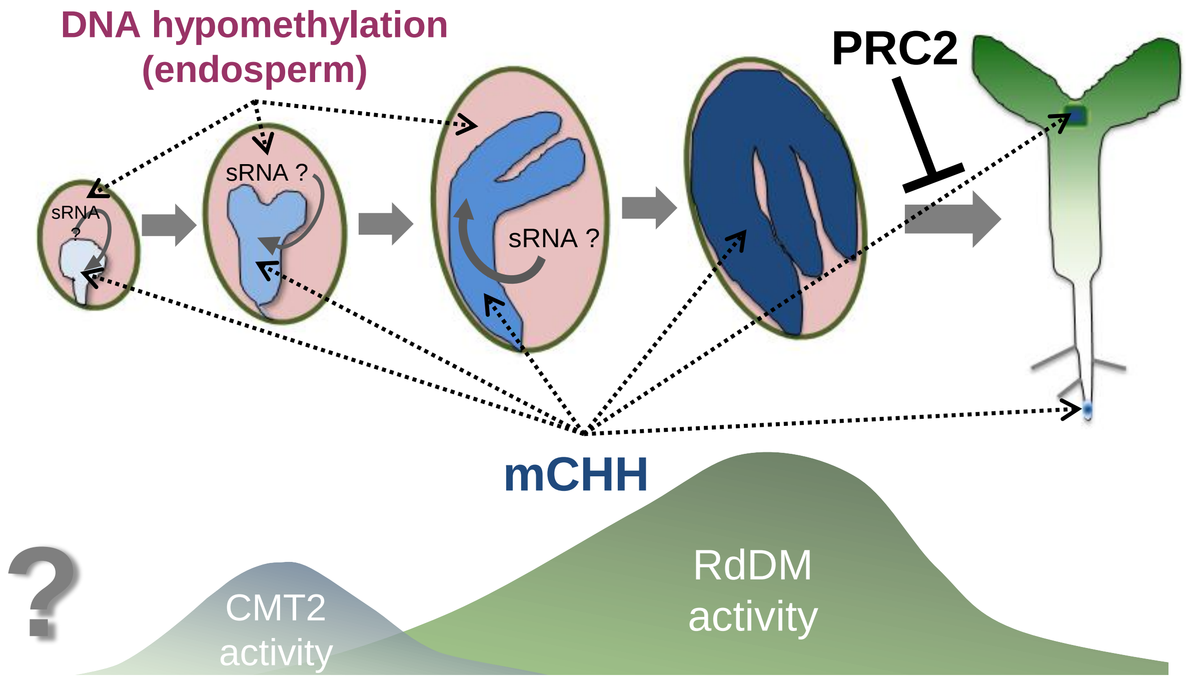

Figure S13. Model describing DNA methylation dynamics during embryogenesis
Based on previous results from other groups [8, 11] and our own observations, we propose a model of gradual re-establishment of DNA methylation patterns. CHH methylation (represented by blue coloration) continues to increase during embryogenesis and shows two waves of establishment, an early one that is CMT2-dependent and RdDM peaking late at embryo maturation. Because CHH methylation can mount up to 100% for thousands of TEs, we assume that all cells are responsive to RdDM in the embryo. This suggests a model in which epigenetic reprogramming is a global process in the embryo that derives from DNA demethylation of the endosperm. Both processes seem to be spatiotemporally uncoupled with CG/CHG hypomethylation in the endosperm preceding the gain in CHH methylation during embryogenesis. This suggests intermediate steps, for instance the production and mobilization of siRNAs from the endosperm to the embryo. After germination, a phase transition controlled by PRC2, RdDM is mainly maintained in the shoot and root meristems.
